# Buffalo Medical College

**Published:** 1851-03

**Authors:** 


*Buffalo Medical College. Commencement.*—The annual commencement in this institution was on the 26th ult. The examination of candidates before the Curators took place in the forenoon, and the degrees were conferred in the afternoon at *Townsend Hall.* The public exercises were opened with prayer by the Rev. Dr. Shelton. In the absence of the Chancellor of the University, His excellency Millard Fillmore, the degrees were conferred by Dr. T. M. Foote, late minister to Bogota. The address to the graduates was delivered by the Dean of the Faculty, Prof. C. B. Coventry. It was a sterling address, replete with sound judicious counsel to those about to enter on the duties and responsibilities of the practice of medicine.

The number of graduates was thirty. We defer the names, for want of space, till the next No.

